# CircAXL Knockdown Alleviates Aβ_1-42_-Induced Neurotoxicity in Alzheimer’s Disease via Repressing PDE4A by Releasing miR-1306-5p

**DOI:** 10.1007/s11064-022-03563-7

**Published:** 2022-03-01

**Authors:** Shengxi Meng, Bing Wang, Wentao Li

**Affiliations:** 1 Department of Traditional Chinese Medicine, Shanghai Jiao Tong University Affiliated Sixth People’s Hospital, No. 600 Yi Shan Road, Xuhui District, Shanghai, 200233 China; 2 Department of Encephalopathy, Shanghai Municipal Hospital of Traditional Chinese Medicine, Shanghai City, China

**Keywords:** circAXL, miR-1306-5p, PDE4A, Alzheimer’s disease, Aβ_1-42_

## Abstract

The development of Alzheimer’s disease (AD) is implicated with the dysregulation of numerous circular RNAs (circRNAs). However, the function of several circRNAs remains unclear. The aim of this study was to investigate the role of circular AXL receptor tyrosine kinase (circAXL) in AD. Cell models of AD were constructed by treating SK-N-SH cells with amyloid-β (Aβ_1-42_). The expression of circAXL, miR-1306-5p and phosphodiesterase 4A (PDE4A) mRNA was detected by quantitative real-time PCR (qPCR). Cell viability was checked by CCK-8 assay. The production of inflammatory factors was monitored by ELISA. Cell apoptosis was checked by flow cytometry assay. Oxidative stress was assessed by ROS level, MDA level and SOD activity using commercial kits. Endoplasmic reticulum (ER) stress was assessed by ER-related protein markers using western blotting. The relationship between miR-1306-5p and circAXL or PDE4A was validated by RIP assay and dual-luciferase reporter assay. Serum exosomes were isolated by centrifugation to assess the diagnostic value of exosomal circAXL, miR-1306-5p and PDE4A. CircAXL was overexpressed in Aβ_1-42_-treated SK-N-SH cells. CircAXL knockdown alleviated Aβ_1-42_-induced cell cytotoxicity, cell apoptosis, inflammation, oxidative stress and endoplasmic reticulum (ER) stress in SK-N-SH cells. MiR-1306-5p was screened as a target of circAXL, and miR-1306-5p inhibition abolished the effects of circAXL knockdown. MiR-1306-5p inhibited the expression of PDE4A, and circAXL regulated PDE4A expression by targeting miR-1306-5p. MiR-1306-5p restoration also alleviated Aβ_1-42_-induced cell injuries, while PDE4A reintroduction abolished the effects of miR-1306-5p restoration. Exosomal circAXL and exosomal miR-1306-5p had diagnostic values for AD. CircAXL knockdown alleviates Aβ_1-42_-induced neurotoxicity in AD pathology via repressing PDE4A by releasing miR-1306-5p.

**Supplementary Information:**

The online version contains supplementary material available at 10.1007/s11064-022-03563-7.

## Introduction

Alzheimer’s disease (AD), a progressive neurodegenerative disease, is the most common form of dementia, characterized by memory impairment and cognitive decline [1]. AD has longer asymptomatic preclinical features, and individuals with normal cognition can also suffer from the disease [2]. It is estimated that the prevalence rate for people over 65 years old is 13%, and the prevalence rate for people over 75 years old is 44% [3]. In AD, the accumulation of β-amyloid (Aβ) may interact with neuronal subcellular organelles, trigger neuronal dysfunction and apoptosis, and lead to memory decline and dementia [1, 4]. Aβ, a proteolytic derivative of the large transmembrane protein APP, particularly the 42-amino-acid form of Aβ (Aβ_1-42_), plays a crucial role in all forms of AD [5]. Therefore, targeted therapy to prevent Aβ-induced neuronal dysfunction is an effective strategy for the treatment of AD.

Circular RNAs (circRNAs) are a group of closed-loop, non-coding RNA molecules, with enormous regulatory potential in human diseases. Unlike linear transcripts, circRNAs express high stability in mammalian cells due to the lack of 5′ cap and 3′ tail [6]. Emerging studies discover that circRNA deregulation is associated with the initiation and development of human neurological diseases [7]. For example, circ_0067835 was involved in temporal lobe epilepsy, and poor circ_0067835 expression was correlated to increased seizure frequency [8]. Besides, circSLC8A1 was overexpressed in Parkinson’s disease, and increased circSLC8A1 was linked to oxidative stress in this disorder [9]. As for AD, Li et al. performed circRNA sequencing and provided numerous circRNAs with aberrant expression in cerebrospinal fluid samples from AD patients [10]. We speculated that the deregulation of numerous circRNAs was probably associated with AD development. CircRNAs are conventionally named by their parental genes [11]. CircLPAR1 (circRNA lysophosphatidic acid receptor 1), circAXL (circRNA AXL receptor tyrosine kinase), circGPHN (circRNA gephyrin), and circGPI (circRNA glucose-6-phosphate isomerase) were shown to be upregulated in cerebrospinal fluid samples from AD patients by a circRNA microarray [10]. We constructed AD cell models by treating SK-N-SH neuron cells with Aβ_1-42_ and examined the expression of these differently expressed circRNAs. The data showed that circAXL was upregulated in Aβ_1-42_-treated SK-N-SH cells with the highest level compared to other circRNAs. However, the role of circAXL was rarely investigated in any human diseases. We thus aimed to determine the function of circAXL in Aβ_1-42_-treated SK-N-SH cells to understand the pathogenesis of AD.

CircRNAs modulate gene expression through multiple putative mechanisms, such as functioning as competing endogenous RNA (ceRNA) to compete with special genes for microRNA (miRNA) binding site [12]. For instance, circHDAC9 acted as miR-138 sponge to promote sirtuin-1 expression, leading to the suppression of Aβ production in AD [13]. Here, miRNAs potentially targeted by circAXL were screened in this study. Besides, we constructed a ceRNA network of circAXL by identifying the target genes that shared the same miRNA binding site with circAXL to address the potential mechanism of circAXL in Aβ_1-42_-induced neurotoxicity.

We ensured the expression level of circAXL in Aβ_1-42_-treated SK-N-SH cells and performed loss-function assays to determine the role of circAXL on Aβ1-42-induced cell cytotoxicity, cell apoptosis, inflammation, oxidative stress and endoplasmic reticulum (ER) stress in SK-N-SH cells. Moreover, we constructed a circAXL/miR-1306-5p/phosphodiesterase 4A (PDE4A) network to understand the mechanism of circAXL in AD.

## Materials and Methods

### Cell Models

SK-N-SH cells were purchased from Procell (Wuhan, China) and cultured in MEM (Procell) containing 10% FBS. Aβ_1-42_ (Sigma, St. Louis, MO, USA) was used to treat SK-N-SH cells (20 μM) for 24 h. Cells in the Control group were not treated (Table 1).

**Table 1:** Clinical features of the patients with AD and normal

	Normal(n = 19)	AD(n = 32)	*P* value
Males/females	9/10	16/16	0.856
Age (mean ± SD)	77.23 ± 5.42	79.35 ± 6.91	0.259
Disease duration (year)	/	4.9 ± 1.7	
MMSE(mean ± SD)	/	14.2 ± 4.8	
Diabetes %(n)	10.53(2)	28(8)	0.208
Hypercholesterolemia %(n)	10.53(2)	21.88(7)	0.304
Hypertension %(n)	31.58(6)	59.37(20)	0.033*
circAXL (mean ± SD)	1 ± 0.25	1.40 ± 0.47	0.001*
miR-1306-5p (mean ± SD)	1 ± 0.33	0.72 ± 0.24	0.001*
PDE4A (mean ± SD)	1 ± 0.32	1.14 ± 0.40	0.201

*AD* Alzheimer's disease, *MMSE* mini-mental state examination

*P < 0.05

### Quantitative Real-Time PCR (qPCR)

Trizol reagent (Solarbio, Beijing, China) was applied to isolate total RNA from cells, according to the manufacturer’s instruction. Then, cDNA was synthesized using ProtoScript® First Strand cDNA Synthesis Kit (New England Biolabs, Beverly, MA, USA) and next quantified using FastStart™ Universal SYBR® Green Master (Sigma). For miRNA, cDNA synthesis and quantification were performed using MicroRNA first-strand synthesis and miRNA quantitation kits (Takara, Dalian, China). β-actin or U6 was used as an internal reference, and the data were calculated using the 2^−ΔΔCt^ method. 3 duplicates were set for each sample in 3 wells, and a total of 3 independent biological experiments were concluded. Primer sequences are described in Table 2.

**Table 2:** Primer sequences used in qPCR

Name	Primer sequences (5′–3′)
*circAXL*	
Forward	CTGGAGGTGGCTTGGACTC
Reverse	GGGGAGCACTGTGATGGT
*PDE4A*	
Forward	CGAAAGGAAGCTGCAGAGCC
Reverse	CAACCTACACCCCGCTCAG
*miR-1306-5p*	
Forward	GCCACCTCCCCTGCAAA
Reverse	AGTGCAGGGTCCGAGGTATT
*GAPDH*	
Forward	ACAGCCTCAAGATCATCAGC
Reverse	GGTCATGAGTCCTTCCACGAT
*U6*	
Forward	CTCGCTTCGGCAGCACATATACT
Reverse	AGTGCAGGGTCCGAGGTATT
*β-actin*	
Forward	ACAATGTGGCCGAGGACTTT
Reverse	GTAACAACGCATCTCATATTTGGAA
*miR-4731-5p*	
Forward	TGCTGGGGGCCACATG
Reverse	AGTGCAGGGTCCGAGGTATT
*miR-6746-5p*	
Forward	ACGCTTCACGAATTTGCGTGTC
Reverse	GCCGGGAGAAGGAGGTG
*miR-3605-3p*	
Forward	CGCCTCCGTGTTACCTGTC
Reverse	AGTGCAGGGTCCGAGGTATT

### RNase R Digestion

Total RNA isolated from cells was digested with RNase R (2 U/μg; Epicentre, Madison, WI, USA) for 30 min at 37 °C. After reverse transcription, qPCR was performed to detect the expression of circAXL and linear GAPDH. A total of 3 independent biological experiments were concluded.

### Subcellular Location

Cytoplasmic RNA and nuclear RNA were separately isolated using the Cytoplasmic & Nuclear RNA Purification Kit (Norgen, Thorold, Canada). The expression levels of circAXL in each section were determined by qPCR, with GAPDH or U6 as the internal reference in cytoplasmic fraction or nuclear fraction, respectively. A total of 3 independent biological experiments were concluded.

### Cell Transfection

CircAXL-specific siRNA (si-circAXL; Genepharma, Shanghai, China) was used for cricAXL knockdown, with si-NC as the negative control. MiR-1306-5p mimic (miR-1306-5p), miR-1306-5p inhibitor (anti-miR-1306-5p) and their negative controls (miR-NC and anti-NC) were all provided by Ribobio (Guangzhou, China). Fusion pcDNA-PDE4A overexpression vector (PDE4A; Genepharma) was used for PDE4A overexpression, with pcDNA vector as the control. SK-N-SH cells were transfected with these vectors or oligonucleotides using the lipofectamine 3000 reagent (Invitrogen, Carlsbad, CA, USA).

### CCK-8 Assay

Cells with transfection were cultured for 24 h and then plated into a 96-well plate (5000 cells per well) and then cultured for 48 h in an incubator at 37 °C. CCK-8 reagent (Invitrogen) was added into each well (10 μL per well) to treat cells for 2 h. Subsequently, the absorbance at 450 nm was examined using a microplate reader (Thermo Fisher Scientific, Waltham, MA, USA). 3 duplicates were set for each sample in 3 wells, and CCK-8 assay was independently performed 3 times.

### ELISA

Cells with various transfections were cultured for 48 h, and cell culture medium was then collected for analysis. To assess the releases of inflammatory factors, human IL-1β ELISA kit (KeyGen, Nanjing, China) and human TNF-α ELISA kit (KeyGen) were enrolled in this study. To assess the level of cyclic adenosine monophosphate (cAMP), cAMP Assay Kit (competitive ELISA) (Abcam) was used in this study. All procedures of ELISA were conducted according to the protocols. Each sample contained 3 repeats in 3 wells, and a total of 3 independent experiments were carried out.

### Flow Cytometry Assay

Cells after treatment or transfection were plated in 6-well plates and cultured for 48 h. Cells were digested with trypsin and washed with PBS. Next, cells were resuspended in Annexin V-FITC binding buffer, followed by the treatment with Annexin V-FITC and propidium iodide from the Annexin V-FITC Apoptosis Detection Kit (Beyotime, Shanghai, China). Afterwards, flow cytometry was conducted to distinguish the apoptotic cells using a flow cytometer. A total of 3 independent experiments were carried out.

### Oxidative Stress Assay

Oxidative stress was assessed according to ROS production, MDA level and SOD activity. These indicators were investigated using the commercial kits purchased from Beyotime (Shanghai, China). Each sample contained 3 repeats in 3 wells, and a total of 3 independent experiments were implemented.

### Western Blotting

ER-related markers, including heat shock protein family A (Hsp70) member 5 (HSPA5, also known as GRP78), DNA Damage Inducible Transcript 3 (DDIT3, also known as CHOP), activating transcription factor 4 (ATF4) and caspase12 (CASP12), were quantified by western blot. The primary antibodies were purchased from Abcam, including anti-HSPA5 (ab21685; Abcam, Cambridge, MA, USA), anti-DDIT3 (ab11419; Abcam), anti-ATF4 (ab184909; Abcam), anti-CASP12 (anti-caspase12; ab62484; Abcam), anti-PDE4A (ab200383; Abcam). Total protein was extracted using RIPA lysis buffer (Beyotime) and quantified by BCA kit (Beyotime). Protein was separated and transferred onto PVDF membranes. Protein on membranes was blocked by 5% skim milk and subsequently incubated with the primary antibodies and the secondary antibody (ab205718; Abcam). Finally, the protein bands were emerged using the ECL reagent (Beyotime) and imaged using Amersham Imager. A total of 3 independent experiments were implemented.

### Pull-Down Assay

Biotin-labeled circAXL probe (5′-TCTTGTTCAGCCCTGCAGGGTGCAG-3′) was directly designed and synthesized by Beyotime and used for circAXL enrichment. Then, probe-coated streptavidin dynabeads (Thermo Fisher Scientific) were prepared for pull-down assay. SK-N-SH cells were lysed, and cell lysates were incubated with the dynabeads. MiRNAs pulled down by circAXL probe were eluted and analyzed by qPCR. 3 independent experiments were performed for this assay.

### RIP Assay

For RIP assay, Magna RIP™ Kit (Millipore Corp, Billerica, MA, USA) was used here. In brief, SK-N-SH cells were lyzed using RIP lysis buffer, and cell lysates were incubated with Protein A/G magnetic beads conjugated with Ago2 antibody (Millipore Corp) or IgG antibody (Millipore Corp). RNA complexes bound to beads were eluted by using Trizol reagent and analyzed by qPCR. 3 independent experiments were performed for this assay.

### Dual-Luciferase Reporter Assay

The binding site between circAXL and miR-1306-5p was predicted by starbase (http://starbase.sysu.edu.cn/), and the binding site between miR-1306-5p and PDE4A 3′UTR was predicted by targetscan (http://www.targetscan.org/vert_72/). According to the sequence of binding site, the mutant-type (MUT) sequences of circAXL and PDE4A 3′UTR were designed. Then, the wild-type (WT) and MUT reporter plasmids of circAXL and PDE4A 3′UTR were constructed, named as WT-circAXL, MUT-circAXL, WT-PDE4A 3′UTR and MUT-PDE4A 3′UTR. For dual-luciferase reporter assay, SK-N-SH cells were transfected with miR-1306-5p and abovementioned reporter plasmid, respectively, with miR-NC as the control. Luciferase activity was examined using the Dual-Luciferase Reporter Assay System (Promega, Madison, WI, USA). 3 independent experiments were performed for this assay.

### Exosome Isolation

The study was authorized by the Ethics Committee of Shanghai Jiao Tong University Affiliated Sixth People’s Hospital. Blood samples were collected from AD patients (n = 32) and healthy controls (n = 19) recruited from Shanghai Jiao Tong University Affiliated Sixth People’s Hospital. The clinical features of these subjects were shown in Table 1. Patients were diagnosed with AD based on the criteria of the National Institute of Neurological and Communication Disorders and Stroke/Alzheimer’s Disease and Related Disorder Association [14]. Healthy controls underwent physical examination were enrolled, and they had no AD or other neurological diseases and malignant tumors. Serum samples were obtained from blood by centrifugation. By using the exoEasy Maxi Kit (QIAGEN, Duesseldorf, Germany), serum-derived exosomes were easily isolated by differential centrifugation. To observe the morphology of exosomes, the isolated exosomes were resuspended in PBS and placed on a formvar carbon-coated copper grid with 0.125% Formvar. The grid was stained with 1% uranyl acetate and washed with PBS. Images were taken using transmission electron microscopy (TEM) (Hitachi, Tokyo, Japan). The size and concentration (participates/mL) of exosomes were identified by nanoparticle tracking analysis (NTA) using NanoSight NS300 instrument (Malvern, Worcestershire, UK) as previously mentioned [15]. Moreover, the existence of exosomes was also characterized by exosome surface markers, including CD81 (anti-CD81, ab109201), CD63 (anti-CD63, ab134045) and TSG101 (anti-TSG101, ab125011). The expression of these markers was ascertained by western blotting as mentioned above.

### Statistical Analysis

GraphPad Prism 7.0 (GraphPad Software, La Jolla, CA, USA) was used for statistical analysis and data processing. Student’s *t*-test or ANOVA (with Tukey’s post-hoc test) was used to analyze the difference in different groups as appropriate. The data were shown as the mean ± SD. *P* < 0.05 indicated statistically significant.

## Results

### The Expression of circAXL was Increased in Aβ_1-42_-Treated SK-N-SH Cells

**Fig. 1:**
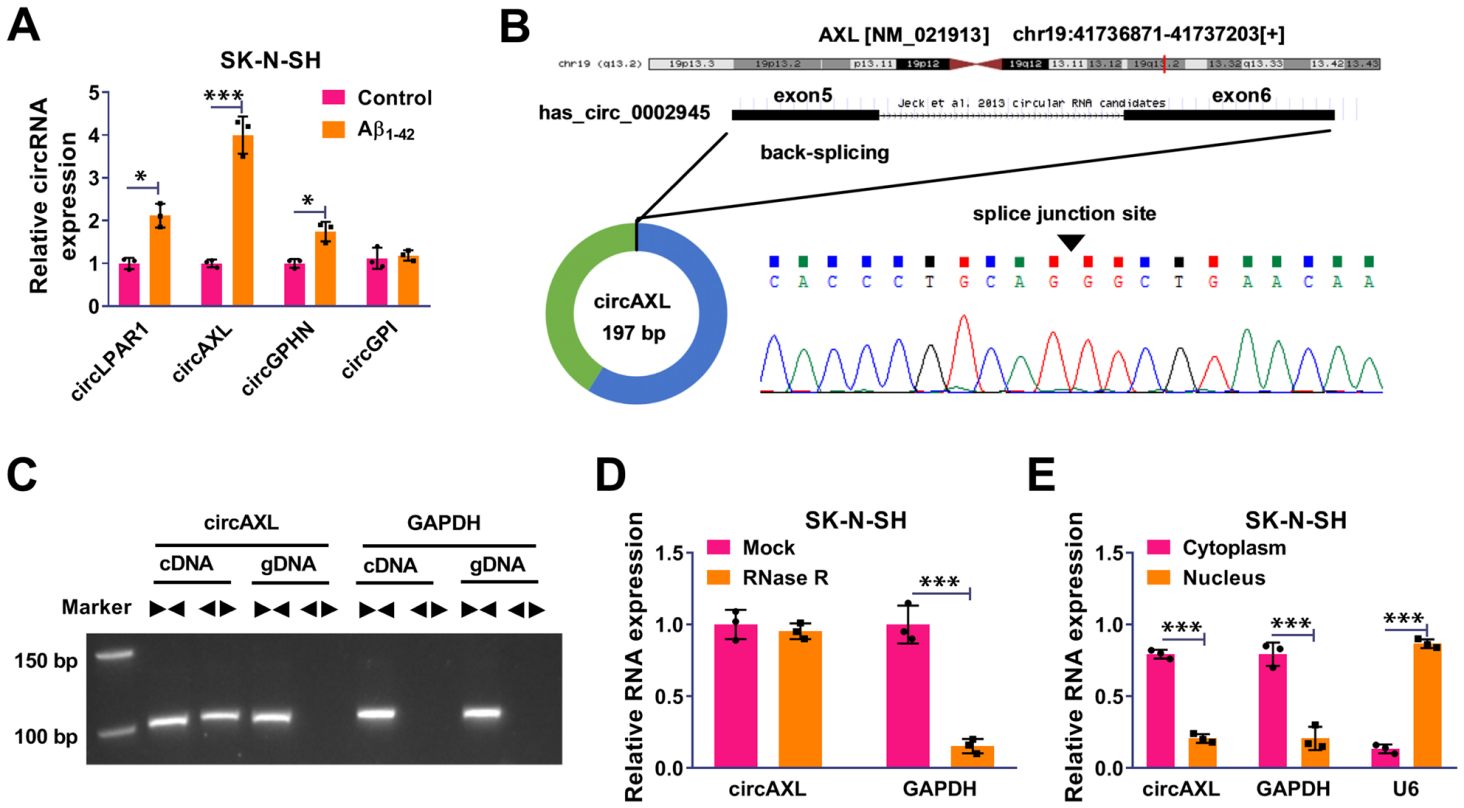
CircAXL expression was elevated in Aβ_1-42_-treated SK-N-SH cells. **A** The expression of screened circRNAs in Aβ_1-42_-treated SK-N-SH cells was detected by qPCR. **B** The detailed information of circAXL. **C** CircAXL was amplified using diverse primers from cDNA to identify the existence of circAXL. **D** The stability of circAXL was checked using RNase R. **E** The distribution of circAXL was checked by qPCR. *P < 0.05, ***P < 0.001; ANOVA (with Tukey’s post-hoc test) was used to analyze the difference

We chose 4 circRNAs that were previously reported to be highly expressed in AD patients [10] and detected their expression levels in Aβ_1-42_-treated SK-N-SH cells. The data showed that circAXL expression was the highest in Aβ_1-42_-treated SK-N-SH cells among all circRNAs (Fig. 1A). CircAXL expression was increased in Aβ_1-42_-treated SK-N-SH cells in a dose-dependent manner (from 0 to 20 μM) (Fig. S1A). CircAXL, also known as circ_0002945, was derived from the exon5 and exon6 regions of AXL (NM_021913) mRNA (Fig. 1B). The data from qPCR showed that circAXL could be amplified by diverse primers from cDNA but not from gDNA (Fig. 1C). Besdies, compared to linear GAPDH molecule, circAXL was resistant to RNase R digestion, suggesting the circularity of circAXL (Fig. 1D). In addition, we found that circAXL was mainly distributed in the cytoplasm but not in the nucleus (Fig. 1E). The data indicated the circularity and stability of circAXL and revealed the high expression of circAXL in Aβ_1-42_-treated SK-N-SH cells.

### CircAXL Knockdown Alleviated Aβ1-42-Induced Cell Cytotoxicity, Cell Apoptosis, Inflammation, Oxidative Stress and Endoplasmic Reticulum (ER) Stress in SK-N-SH Cells

**Fig. 2:**
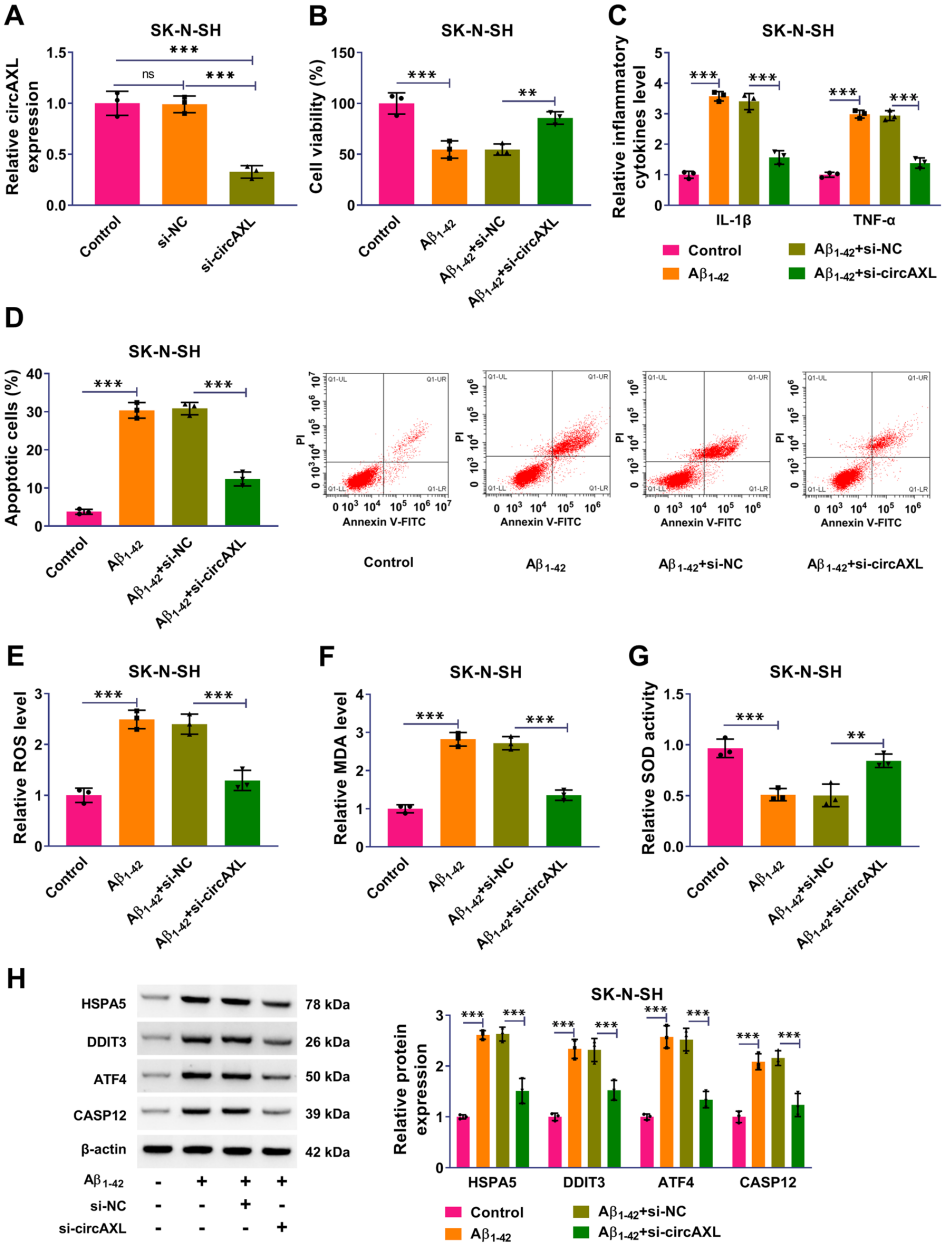
CircAXL knockdown alleviated Aβ_1-42_-induced SK-N-SH cell injuries and dysfunctions. **A** The efficiency of circAXL silencing in SK-N-SH cells was checked by qPCR. **B** The role of circAXL knockdown on cell viability was detected by CCK-8 assay. **C** The role of circAXL knockdown on inflammatory responses was determined by ELISA. **D** The role of circAXL knockdown on cell apoptosis was checked by flow cytometry assay. **E**–**G** The levels of ROS, MDA and SOD were examined to assess oxidative stress. **H** The protein levels of HSPA5, DDIT3, ATF4 and CASP12 were determined by western blotting to assess ER stress. **P < 0.01, ***P < 0.001. ANOVA (with Tukey’s post-hoc test) was used to analyze the difference

The expression of circAXL was markedly declined in Aβ_1-42_-treated SK-N-SH cells with the transfection of si-circAXL (Fig. 2A). The results from CCK-8 assay presented that Aβ1-42-depleted cell viability was largely recovered by the knockdown of circAXL (Fig. 2B). ELISA showed that Aβ_1-42_ triggered the releases of IL-1β and TNF-α in SK-N-SH cells, while the transfection of si-circAXL partly alleviated the releases of these inflammatory cytokines (Fig. 2C). Flow cytometry assay showed that Aβ1-42-induced cell apoptosis was largely alleviated by circAXL knockdown (Fig. 2D). In addition, ROS level and MDA level were increased, while SOD activity was declined in Aβ_1-42_-treated SK-N-SH cells. However, circAXL knockdown repressed ROS level and MDA level and recovered SOD activity (Fig. 2E–G). Additionally, some proteins closely related to ER stress [16] were quantified by western blot. The protein levels of HSPA5, DDIT3, ATF4 and CASP12 largely promoted by Aβ_1-42_ treatment were partly reduced by circAXL downregulation (Fig. 2H). These findings manifested that Aβ1-42-induced cell cytotoxicity, cell apoptosis, inflammation, oxidative stress and ER stress in SK-N-SH cells were alleviated by circAXL knockdown.

### MiR-1306-5p was Screened as a Target of circAXL

**Fig. 3:**
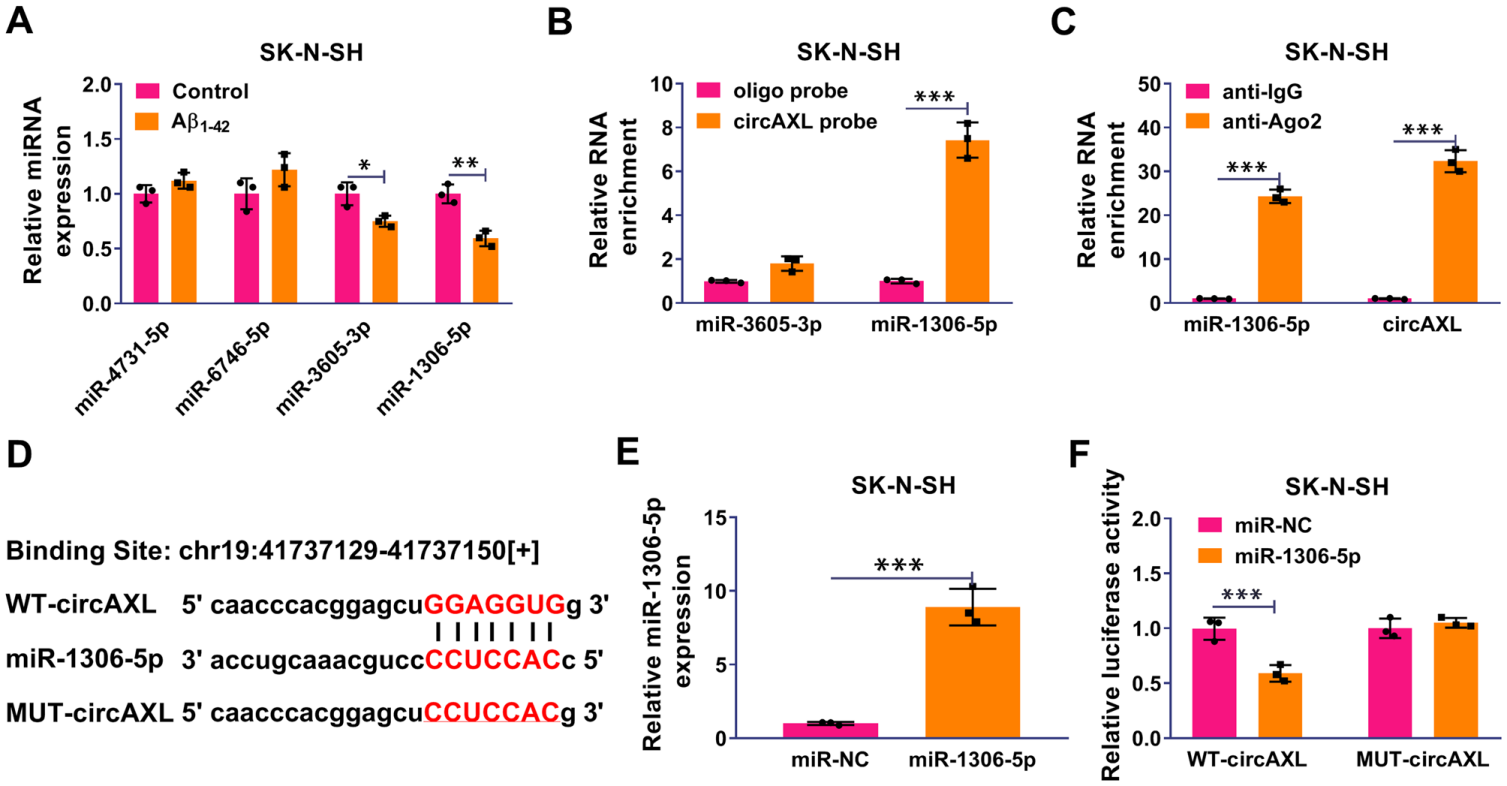
MiR-1306-5p was a target of circAXL. **A** The expression of predicted miRNAs in Aβ_1-42_-treated SK-N-SH cells was detected by qPCR. **B** Pull-down by circAXL probe was used to screen target miRNAs. **C** The binding relationship between circAXL and miR-1306-5p was verified by RIP assay. **D** The WT and MUT sequence fragments of circAXL. **E** The efficiency of miR-1306-5p mimic was checked by qPCR. **F** The binding relationship between circAXL and miR-1306-5p was verified by dual-luciferase reporter assay. ***P < 0.001. Student’s t-test or ANOVA (with Tukey’s post-hoc test) was used to analyze the difference

Bioinformatics database predicted several target miRNAs of circAXL, and 4 miRNAs (miR-4731-5p, miR-6746-5p, miR-3605-3p and miR-1306-5p) were shown to have binding sites with circAXL. We examined their expression in Aβ_1-42_-treated SK-N-SH cells and found that miR-3605-3p and miR-1306-5p were downregulated in Aβ_1-42_-treated SK-N-SH cells (Fig. 3A). Pull-down assay presented that the abundance of miR-1306-5p pulled down by circAXL probe was relatively higher than miR-3605-3p (Fig. 3B). Ago2 binding protein is the core effector of miRNA-mediated RNA-induced silencing complex (RISC). RIP assay manifested that miR-1306-5p and circAXL were significantly enriched in the anti-Ago2 RIP group compared with that in the anti-IgG RIP group (Fig. 3C). Moreover, the wild-type and mutant-type reporter plasmids of circAXL were constructed for dual-luciferase reporter assay (Fig. 3D). MiR-1306-5p expression was reduced in Aβ_1-42_-treated SK-N-SH cells in a dose-dependent manner (Fig. S1B). The transfection of miR-1306-5p notably enriched the expression of miR-1306-5p in SK-N-SH cells (Fig. 3E). Dual-luciferase reporter assay presented that miR-1306-5p transfected with WT-circAXL markedly lessened luciferase activity in SK-N-SH cells (Fig. 3F). The evidence verified that miR-1306-5p was a target of circAXL.

### CircAXL Knockdown Alleviated Aβ_1-42_-Induced Cell Dysfunctions of SK-N-SH by Targeting miR-1306-5p

**Fig. 4:**
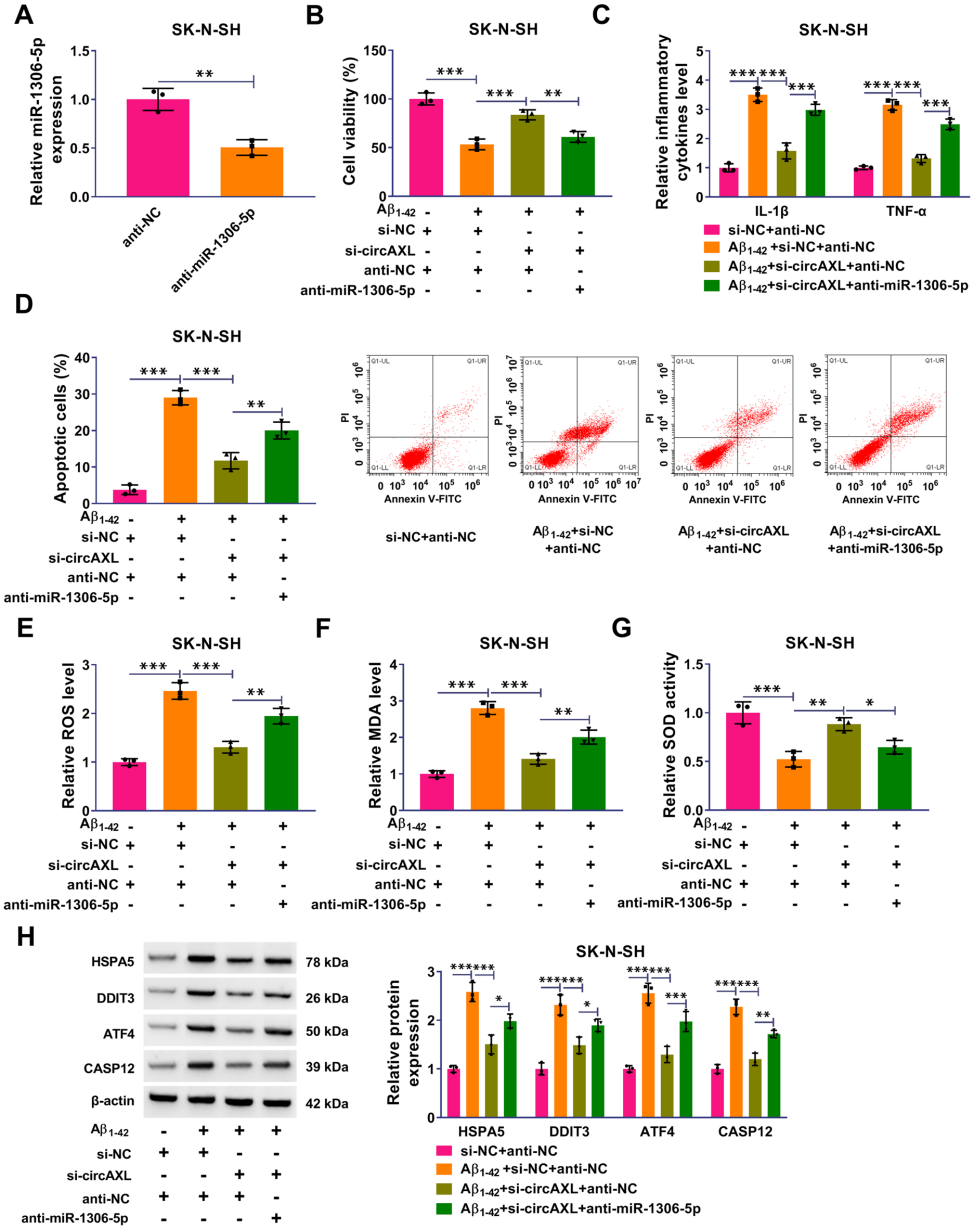
CircAXL played functions partly by targeting miR-1306-5p. **A** The efficiency of miR-1306-5p inhibitor was checked by qPCR. In Aβ_1-42_-treated SK-N-SH cells transfected with si-NC + anti-NC, si-circAXL + anti-NC or si-circAXL + anti-miR-1306-5p, **B** cell viability was checked by CCK-8 assay. **C** Inflammatory response was monitored by ELISA. **D** Cell apoptosis was examined using flow cytometry assay. **E**–**G** ROS level, MDA level and SOD activity were examined using commercial kits. **H** The protein levels of HSPA5, DDIT3, ATF4 and CASP12 were detected by western blotting. *P < 0.05, **P < 0.01, ***P < 0.001. Student’s *t*-test or ANOVA (with Tukey’s post-hoc test) was used to analyze the difference

The transfection of anti-miR-1306-5p significantly reduced the expression of miR-1306-5p in SK-N-SH cells (Fig. 4A). In function, Aβ_1-42_-depleted cell viability of SK-N-SH cells was recovered by circAXL knockdown but repressed by further miR-1306-5p repression (Fig. 4B). Aβ_1-42_-induced the releases of IL-1β and TNF-α were notably ameliorated by circAXL knockdown but restored by miR-1306-5p inhibition (Fig. 4C). Aβ_1-42_-induced SK-N-SH cell apoptosis was also notably ameliorated by circAXL knockdown but promoted by miR-1306-5p inhibition (Fig. 4D). In addition, the levels of ROS and MDA in Aβ_1-42_-treated SK-N-SH cells were attenuated by circAXL knockdown but restored by additional miR-1306-5p absence, and the activity of SOD strengthened by circAXL knockdown was largely repressed by miR-1306-5p absence (Fig. 4E–G). Moreover, the protein levels of HSPA5, DDIT3, ATF4 and CASP12 were repressed in Aβ_1-42_-treated SK-N-SH cells after circAXL knockdown, while further miR-1306-5p suppression partly restored their expression levels (Fig. 4H). These data manifested that circAXL knockdown alleviated Aβ_1-42_-induced cell dysfunctions of SK-N-SH by targeting miR-1306-5p.

### PDE4A was a Target of miR-1306-5p

**Fig. 5:**
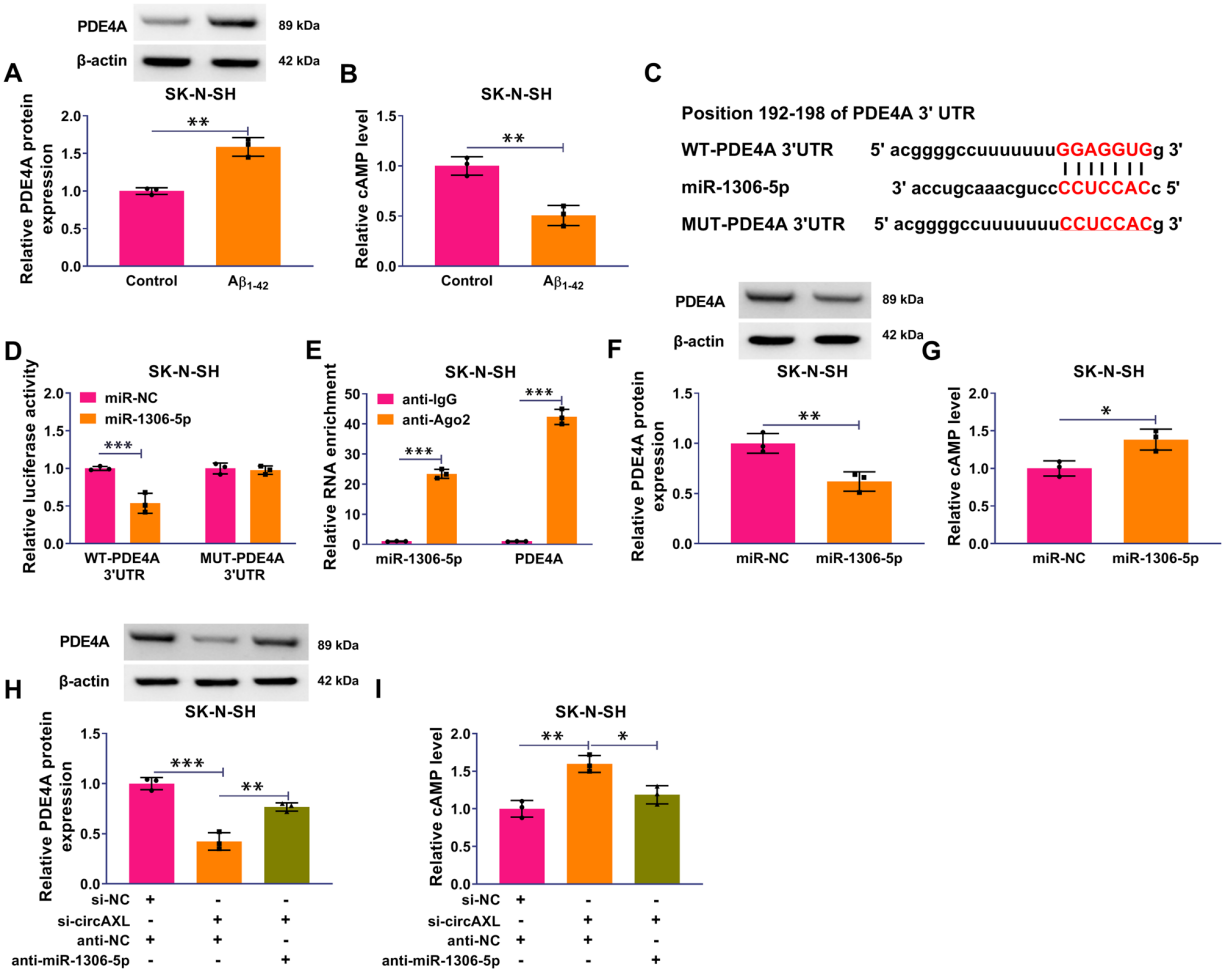
PDE4A was a target of miR-1306-5p. **A** The protein level of PDE4A in Aβ_1-42_-treated SK-N-SH cells was detected by western blotting. **B** The level of cAMP in Aβ_1-42_-treated SK-N-SH cells was detected by ELISA. **C** and **D** The target relationship between miR-1306-5p and PDE4A was validated by dual-luciferase reporter assay. **E** The target relationship between miR-1306-5p and PDE4A was validated by RIP assay. **F** The protein level of PDE4A in SK-N-SH cells with miR-1306-5p restoration was detected by western blotting. **G** The level of cAMP in SK-N-SH cells transfected with miR-1306-5p was detected by ELISA. **H** The protein level of PDE4A in SK-N-SH cells transfected with si-NC + anti-NC, si-circAXL + anti-NC or si-circAXL + anti-miR-1306-5p was detected by western blotting. **I** The level of cAMP in SK-N-SH cells transfected with si-NC + anti-NC, si-circAXL + anti-NC or si-circAXL + anti-miR-1306-5p was detected by ELISA. *P < 0.05, **P < 0.01, ***P < 0.001. Student’s *t*-test or ANOVA (with Tukey’s post-hoc test) was used to analyze the difference

Among the target genes of miR-1306-5p predicted by targetscan, we found that PDE4A, a key regulator of cAMP degradation rate, was notably upregulated in Aβ_1-42_-treated SK-N-SH cells, in a dose-dependent manner (Fig. 5A and Fig. S1C). The data from ELISA showed that cAMP level was notably decreased in Aβ_1-42_-treated SK-N-SH cells (Fig. 5B). Dual-luciferase reporter assay showed that the cotransfection of miR-1306-5p and WT-PDE4A 3′UTR but not MUT-PDE4A 3’UTR significantly reduced luciferase activity in SK-N-SH cells (Fig. 5C and D). Besides, miR-1306-5p and PDE4A were strikingly enriched in the anti-Ago2 group but not anti-IgG group in RIP assay (Fig. 5E). The enrichment of miR-1306-5p notably suppressed the protein level of PDE4A (Fig. 5F). The level of cAMP was notably increased in SK-N-SH cells after miR-1306-5p restoration (Fig. 5G). Moreover, the data presented that the expression of PDE4A protein was notably inhibited by circAXL knockdown but partly recovered by miR-1306-5p inhibition (Fig. 5H). The level of cAMP was markedly enhanced in SK-N-SH cells after circAXL knockdown but partly repressed by further miR-1306-5p inhibition (Fig. 5I). The data suggested that PDE4A was a target of miR-1306-5p.

### PDE4A Overexpression Partly Reversed the Effects of miR-1306-5p on Aβ_1-42_-Induced Cell Dysfunctions of SK-N-SH

**Fig. 6:**
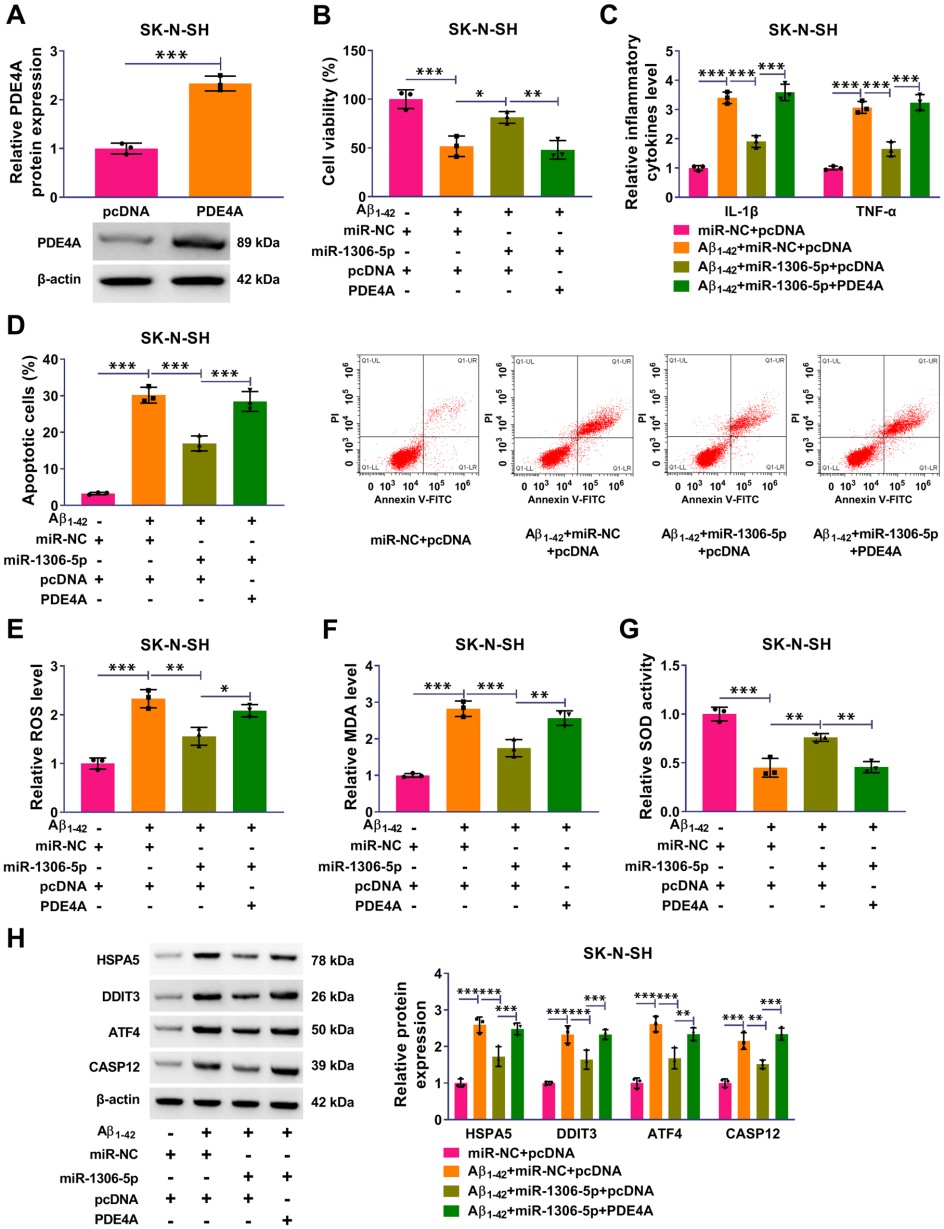
PDE4A overexpression partly reversed the effects of miR-1306-5p on Aβ1-42-induced SK-N-SH cell dysfunctions. **A** The efficiency of PDE4A overexpression was checked by western blotting. In Aβ_1-42_-treated SK-N-SH cells transfected with miR-NC + pcDNA, miR-1306-5p + pcDNA or miR-1306-5p + PDE4A. **B** cell viability was checked by CCK-8 assay. **C** Inflammatory response was monitored by ELISA. **D** Cell apoptosis was examined using flow cytometry assay. **E**–**G** ROS level, MDA level and SOD activity were examined using commercial kits. **H** The protein levels of HSPA5, DDIT3, ATF4 and CASP12 were detected by western blotting. *P < 0.05, **P < 0.01, ***P < 0.001. Student’s *t*-test or ANOVA (with Tukey’s post-hoc test) was used to analyze the difference

The expression of PDE4A was markedly enhanced in SK-N-SH cells transfected with PDE4A compared to pcDNA (Fig. 6A). In function, Aβ_1-42_-impaired cell viability of SK-N-SH cells was restored by miR-1306-5p overexpression but repressed by PDE4A reintroduction (Fig. 6B). The releases of IL-1β and TNF-α stimulated by Aβ_1-42_ were notably ameliorated by miR-1306-5p overexpression but recovered by PDE4A reintroduction (Fig. 6C). Aβ_1-42_-induced SK-N-SH cell apoptosis was also notably ameliorated by miR-1306-5p restoration but promoted by PDE4A overexpression (Fig. 6D). The change of ROS level, MDA level and SOD activity indicated that Aβ_1-42_-induced oxidative stress in SK-N-SH cells was strikingly repressed by miR-1306-5p restoration but largely recovered by PDE4A overexpression (Fig. 6E–G). Moreover, the protein levels of HSPA5, DDIT3, ATF4 and CASP12 were reduced in Aβ_1-42_-treated SK-N-SH cells with miR-1306-5p restoration but largely recovered in Aβ_1-42_-treated SK-N-SH cells with miR-1306-5p restoration plus PDE4A overexpression (Fig. 6H). These data manifested that miR-1306-5p restoration alleviated Aβ1-42-induced cell dysfunctions of SK-N-SH by suppressing PDE4A.

### Exosomal circAXL and miR-1306-5p had Diagnostic Value for AD

**Fig. 7:**
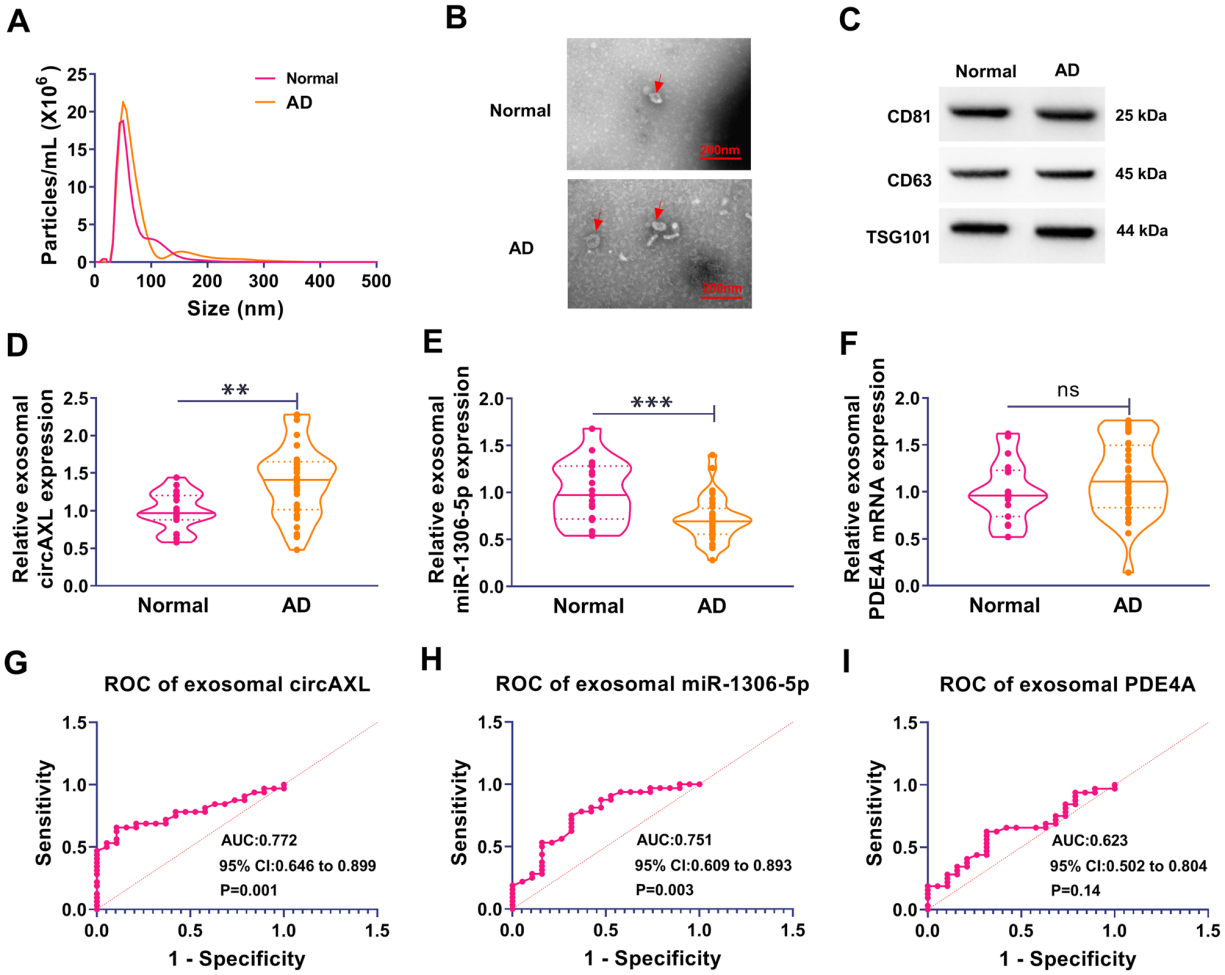
The diagnostic value of exosomal circAXL and miR-1306-5p. **A** The size of exosomes was analyzed by NTA. **B** The morphology of exosomes was observed by TEM. **C** The expression of CD81, CD63 and TSG101 was monitored by western blot. **D**–**F** The expression levels of circAXL, miR-1306-5p and PDE4A mRNA in exosomes from AD serum and normal serum were detected by qPCR. **G**–**I** ROC curve was depicted to analyze the diagnostic value of circAXL, miR-1306-5p and PDE4A. **P < 0.01, ***P < 0.001, *ns* no significance. Student’s *t*-test (unpaired) was used to analyze the difference

We isolated exosomes from serum samples from AD patients and normal controls. The data in Fig. 7A suggested that the size of exosomes mainly distributed from 30 to 200 nm. Representative lipid bilayer structure was observed in the isolated exosomes (Fig. 7B). Exosomal surface markers, including CD81, CD63 and TSG101, were abundantly identified in exosomes (Fig. 7C). The data from qPCR revealed that the expression of circAXL was strikingly increased, while the expression of miR-1306-5p was remarkably decreased in exosomes from AD serum samples compared with that from normal controls (Fig. 7D and E). However, the expression of PDE4A mRNA in different samples had no difference (Fig. 7F). Moreover, receiver operating characteristic (ROC) curve analysis of exosomal circAXL and exosomal miR-1306-5p suggested that exosomal circAXL and miR-1306-5p had diagnostic value for AD (*P* < 0.05; Fig. 7G and H). However, exosomal PDE4A had no diagnostic value for AD (*P* > 0.05; Fig. 7I). The data mainly highlighted that exosomal circAXL and exosomal miR-1306-5p might be used as indicators for AD detection.

## Discussion

Our study investigated the role of circAXL, which was previously shown to be upregulated in cerebrospinal fluid from AD patients [10]. The results mainly discovered that the knockdown of circAXL largely inhibited Aβ_1-42_-induced neuron injuries, including cell cytotoxicity, cell apoptosis, inflammation, oxidative stress and ER stress in SK-N-SH cells. We identified that miR-1306-5p was a target of circAXL, and circAXL shared the same miR-1306-5p binding site with PDE4A 3’UTR. CircAXL downregulation relieved the inhibition on miR-1306-5p and thus decreased the expression of PDE4A. Thus, we proposed that circAXL participated in Aβ_1-42_-induced neuron injuries by targeting the miR-1306-5p/PDE4A axis.

Recent studies have addressed the functional effects of several circRNAs in AD models [13, 17]. For example, circ_0000950 upregulation accelerated neuron apoptosis, promoted inflammatory responses and suppressed neurite outgrowth in Aβ_1-42_-treated PC12 cells [17]. Similarly, we found that Aβ_1-42_ triggered a series of neuronal injuries, such as neuronal cytotoxicity, neuronal apoptosis, inflammation and oxidative stress, while circAXL downregulation largely ameliorated these injuries. Studies showed that activated ER stress promoted the activation of unfolded protein response (UPR), a signal transduction pathway that triggered apoptosis of irreversibly damaged cells [18]. Our data also noticed that Aβ_1-42_ induced ER stress in SK-N-SH cells, while circAXL knockdown alleviated Aβ_1-42_-induced ER stress. Exosomes have gained increasing attention in the biomarker discovery field, and exosomes, as fluid biomarkers, have an enormous advantage to monitor neuronal functions in AD [19]. Exosomes, derived biofluids, carrying candidate protein or non-coding RNA molecules, have diagnostic and therapeutic potency in the clinical practice of AD [20]. We isolated serum-derived exosomes from AD patients and normal subjects and found that circAXL was upregulated, while miR-1306-5p was downregulated in serum-derived exosomes from AD patients. Liu et al. defined that circ_0003391 was a promising biomarker in peripheral blood from AD patients according to the ROC curve analysis [21]. We also performed ROC curve analysis and found that exosomal circAXL and exosomal miR-1306-5p had the potential diagnostic value for AD, while exosomal PDE4A had no noticeable value. These findings strongly supported that exosomal circAXL and exosomal miR-1306-5p were biomarkers for the diagnosis of AD.

MiR-1306-5p was identified to be a target of circAXL. By reviewing the previous studies, we found that miR-1306-5p was proposed to be associated with AD because a total of twelve predicted target genes of miR-1306-5p were involved in processes of AD [22]. Besides, a previous study revealed that the expression of miR-1306-5p was remarkably declined in AD patients [23]. Consistent with these opinions, we found that miR-1306-5p expression was strikingly reduced in Aβ_1-42_-treated SK-N-SH cells. In function, the inhibition of miR-1306-5p reversed the effects of circAXL knockdown and thus recovered Aβ_1-42_-induced SK-N-SH injuries, suggesting that circAXL mediated neuron injuries by targeting miR-1306-5p. The restoration of miR-1306-5p largely alleviated Aβ_1-42_-induced cell cytotoxicity, cell apoptosis, inflammation, oxidative stress and ER stress in SK-N-SH cells, indicating that miR-1306-5p played a protecting role against AD.

Cyclic AMP (cAMP) is a core component of intracellular signaling pathways that regulate a variety of biological functions, including memory, and cAMP enhancers have been regarded as promising therapeutic agents for AD [24]. Phosphodiesterase has long been considered as a target for the treatment of Alzheimer’s disease (AD) [25]. Phosphodiesterase plays a vital role in regulating the degradation rate of cAMP, which has been to be implicated in AD pathogenesis [26]. PDE4A is a member of phosphodiesterase family, also known as cAMP-specific 3′, 5′-PDE4A. Here, we found that circAXL shared the same miR-1306-5p binding site with PDE4A 3′UTR, and circAXL positively regulated PDE4A expression by targeting miR-1306-5p. The expression of PDE4A was strikingly enhanced in Aβ_1-42_-treated SK-N-SH cells, and the data from ELISA showed that Aβ_1-42_ treatment reduced the level of cAMP in SK-N-SH cells. We speculated that the low level of cAMP in Aβ_1-42_-treated SK-N-SH cells was associated with high PDE4A expression. Further analysis discovered that circAXL knockdown enhanced the level of cAMP, while miR-1306-5p inhibition repressed the level of cAMP, indicating that circAXL might regulatePDE4A expression by targeting miR-1306-5p, thus affecting cAMP activity. In function, PDE4A reintroduction reversed the role of miR-1306-5p restoration and thus recovered Aβ_1-42_-induced SK-N-SH cell injuries, which was consistent with the previous findings [27, 28].

There are some limitations to our current work. For example, no data are available on the expression of circAXL, miR-1306-5p and PDE4A in clinical brain specimens of AD subjects, which possibly weakens the clinical implications of these indicators in AD. These issues should be addressed in future work.

## Conclusion

CircAXL was overexpressed in Aβ_1-42_-treated SK-N-SH cells, and high circAXL expression was closely associated with Aβ_1-42_-induced SK-N-SH cell injuries. CircAXL downregulation alleviated Aβ1-42-induced SK-N-SH cell cytotoxicity, cell apoptosis, inflammation, oxidative stress and ER stress partly by enriching miR-1306-5p in turn promoting the inhibition of miR-1306-5p on PDE4A. Besides, exosomal circAXL and exosomal miR-1306-5p could be used as diagnostic markers for AD. Our study for the first time partly determine the role of circAXL in AD cell models, and the circAXL/miR-1306-5p/PDE4A was firstly proposed in our study. These findings provided more insights into the understanding of AD pathogenesis.

## Supplementary Information

Below is the link to the electronic supplementary material.

Supplementary file1 (PDF 2149 kb)

Fig. S1 The expression of circAXL, miR-1306-5p and PDE4A in Aβ1-42-treated SK-N-SH cells. (A) The expression of circAXL was increased in Aβ1-42-treated SK-N-SH cells in a dose-independent manner. (B) The expression of miR-1306-5p was reduced in Aβ1-42-treated SK-N-SH cells in a dose-independent manner. (C) The expression of PDE4A was enhanced in Aβ1-42-treated SK-N-SH cells in a dose-independent manner. *P<0.05, **P<0.01, ***P<0.001. ANOVA (with Tukey’s post-hoc test) was used to analyze the difference. Three independent experiments for these assays were conducted (TIF 374 kb)

## Data Availability

Please contact the correspondence author for the data request.
