# Supplementary material for: CircAXL Knockdown Alleviates Aβ1-42-Induced Neurotoxicity in Alzheimer’s Disease via Repressing PDE4A by Releasing miR-1306-5p
Source: Neurochem Res. 2022 Mar 1;47(6):1707–20. doi: 10.1007/s11064-022-03563-7 (PMC9124172; doi:10.1007/s11064-022-03563-7)

Fig2H

1 Control

2 Aβ_{1-42}

3 Aβ_{1-42}+si-NC

4 Aβ_{1-42}+si-circAXL

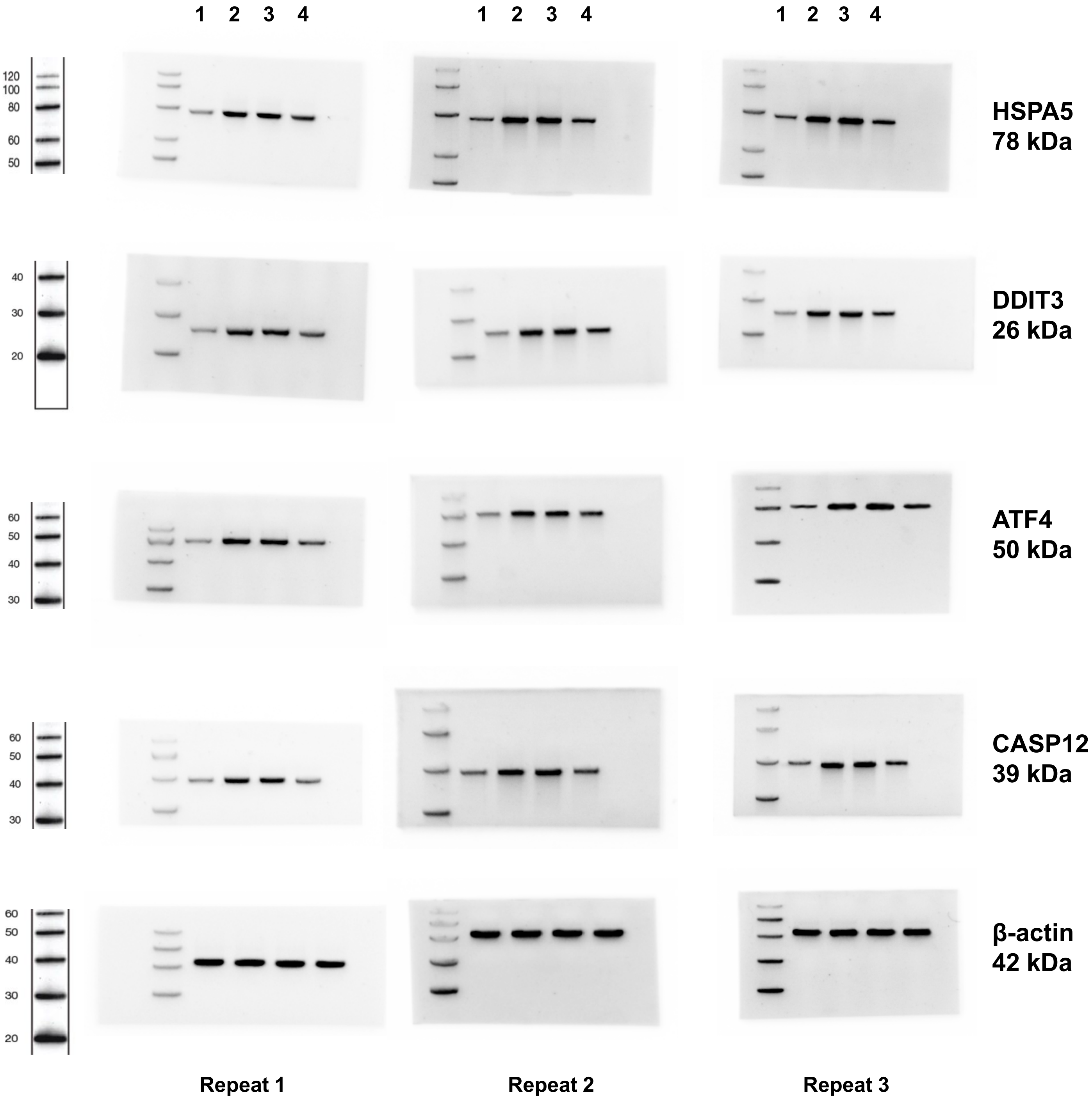

Fig4H

1 si-NC+anti-NC

2 Aβ_{1-42}+si-NC+anti-NC

3 Aβ_{1-42}+si-circAXL+anti-NC

4 Aβ_{1-42}+si-circAXL+anti-miR-1306-5p

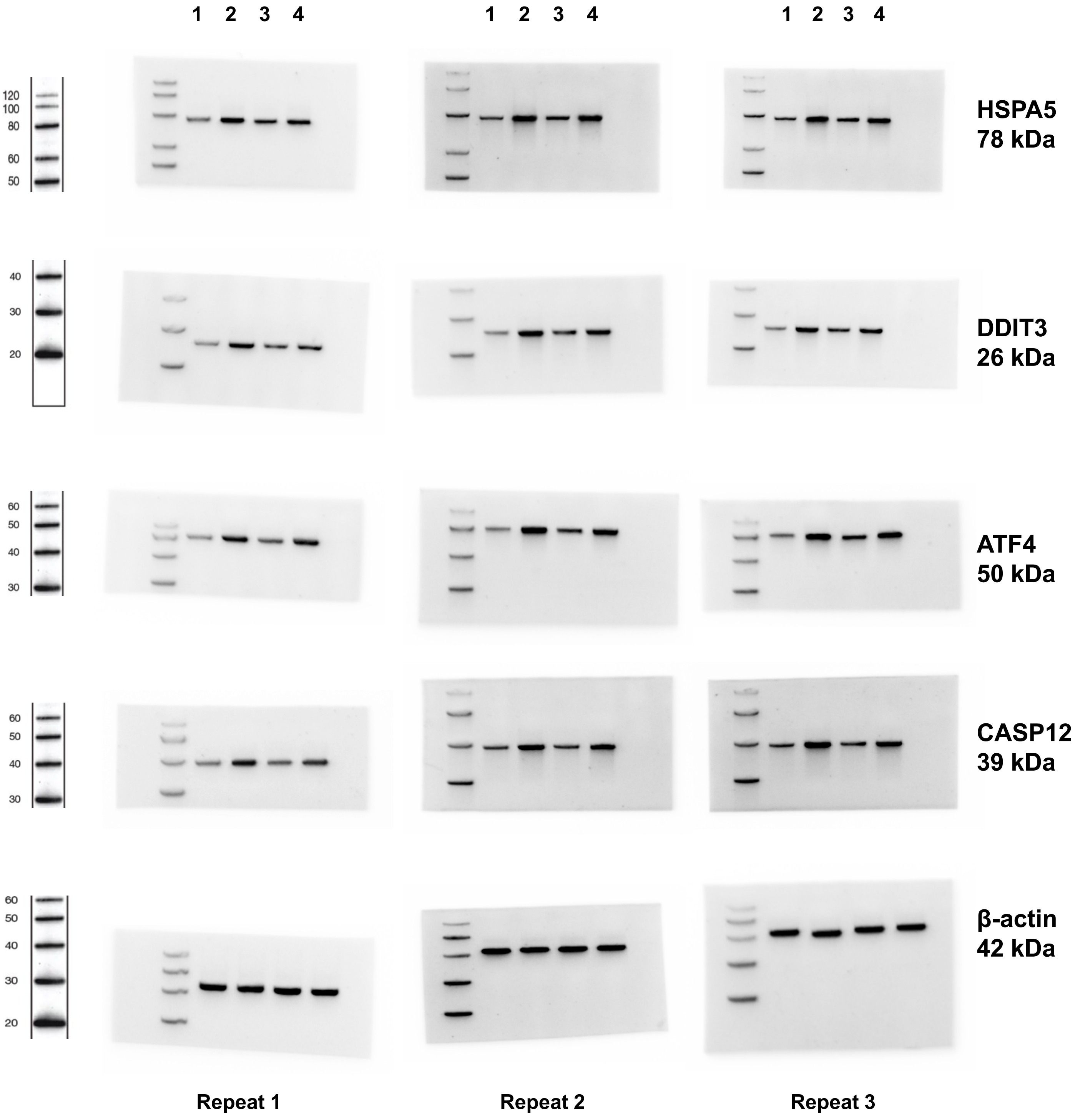

Fig5A 1 Control 2 A$\beta_{1\text{-}42}$

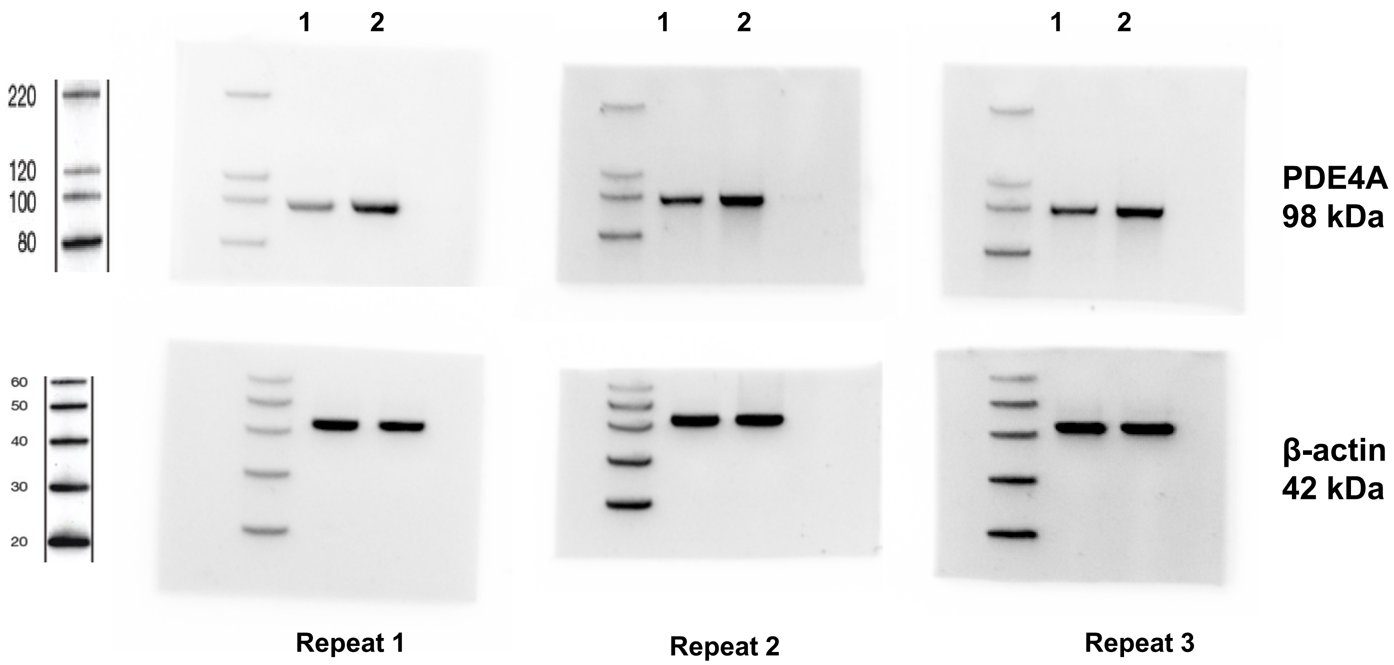

Fig5F 1 miR-NC 2 miR-1306-5p

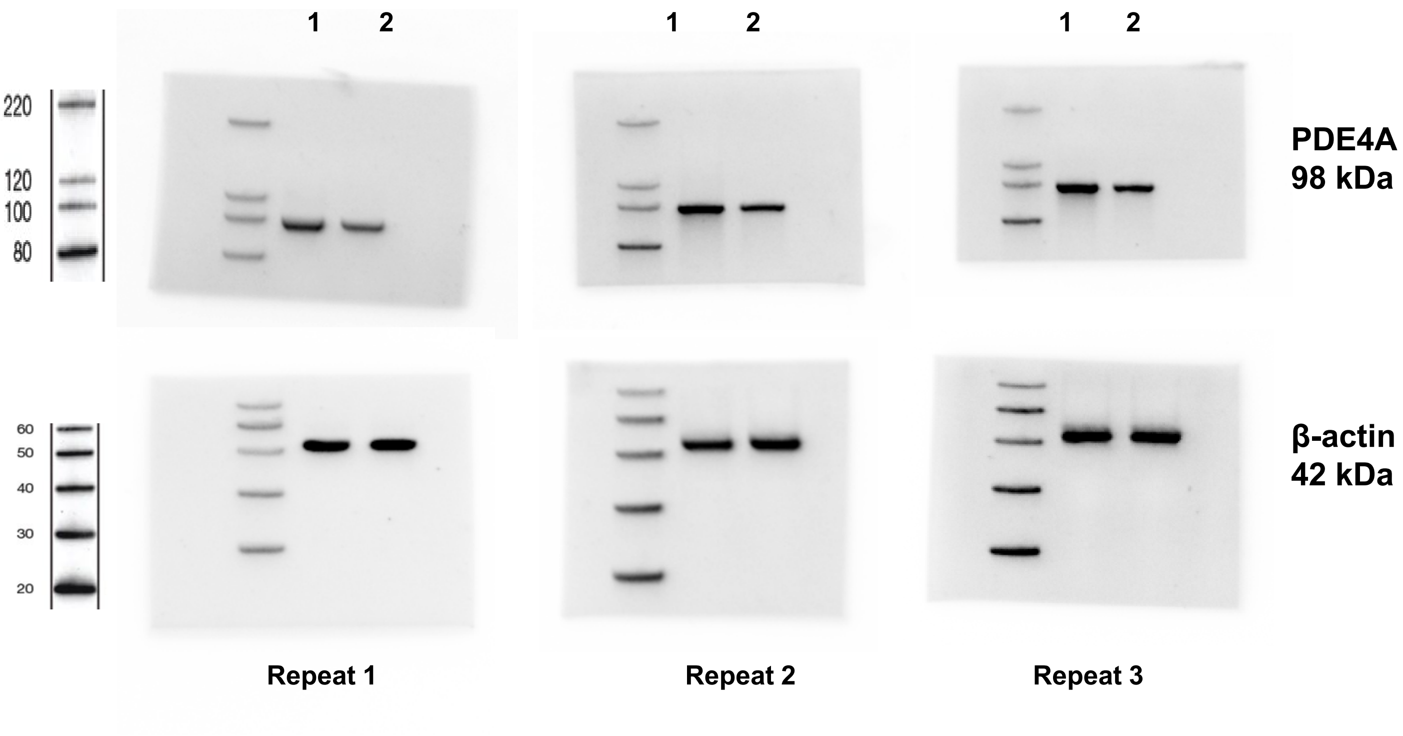

Fig5H

1 si-NC+anti-NC

2 si-circAXL+anti-NC

3 si-circAXL+anti-miR-1306-5p

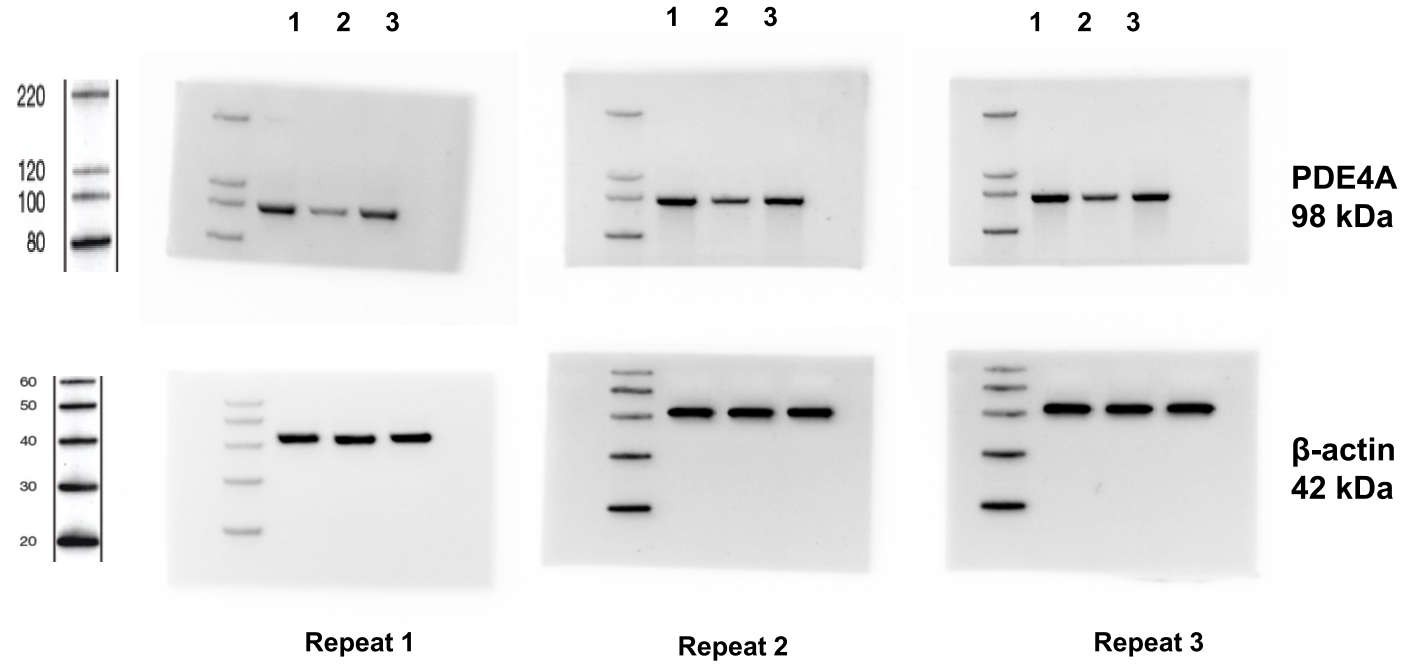

Fig6A

1 pcDNA 2 PDE4A

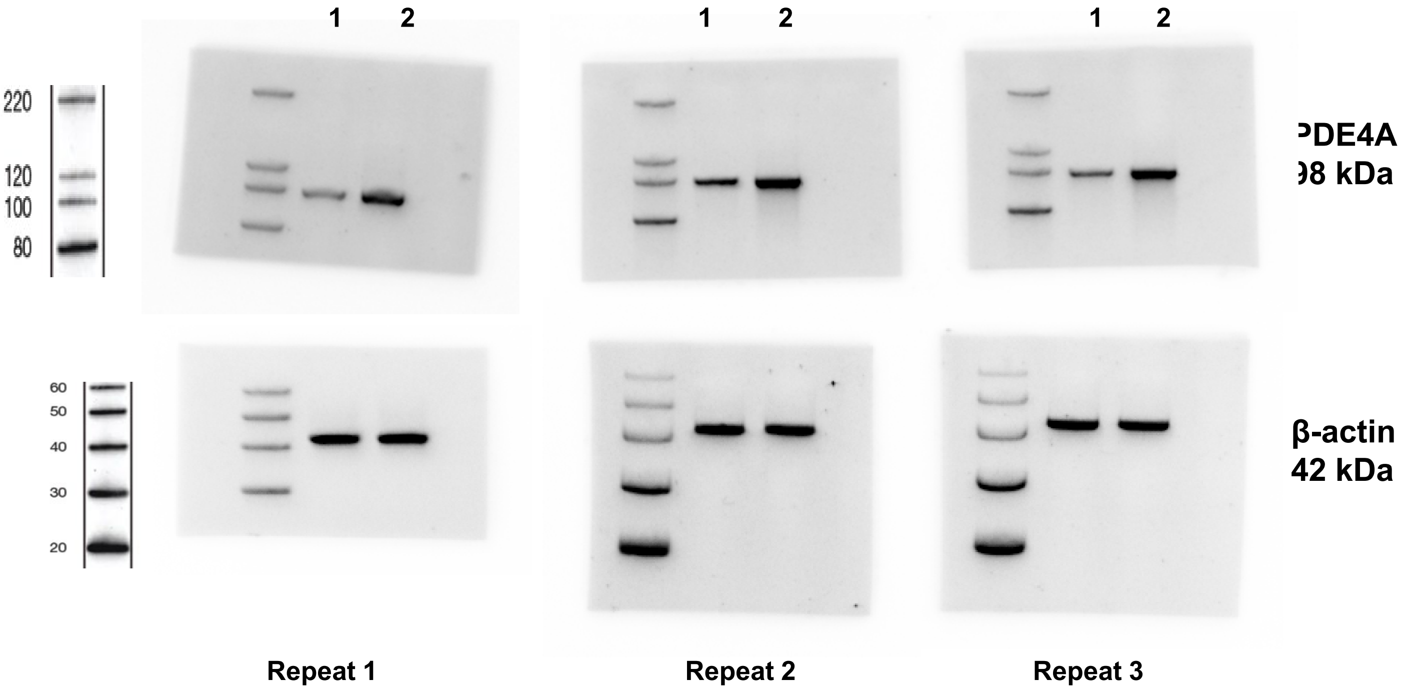

Fig6H

1 miR-NC+pcDNA

2 $A\beta_{1\text{-}42}$+miR-NC+pcDNA

3 $A\beta_{1\text{-}42}$+miR-1306-5p+pcDNA

4 $A\beta_{1\text{-}42}$+miR-1306-5p+PDE4A

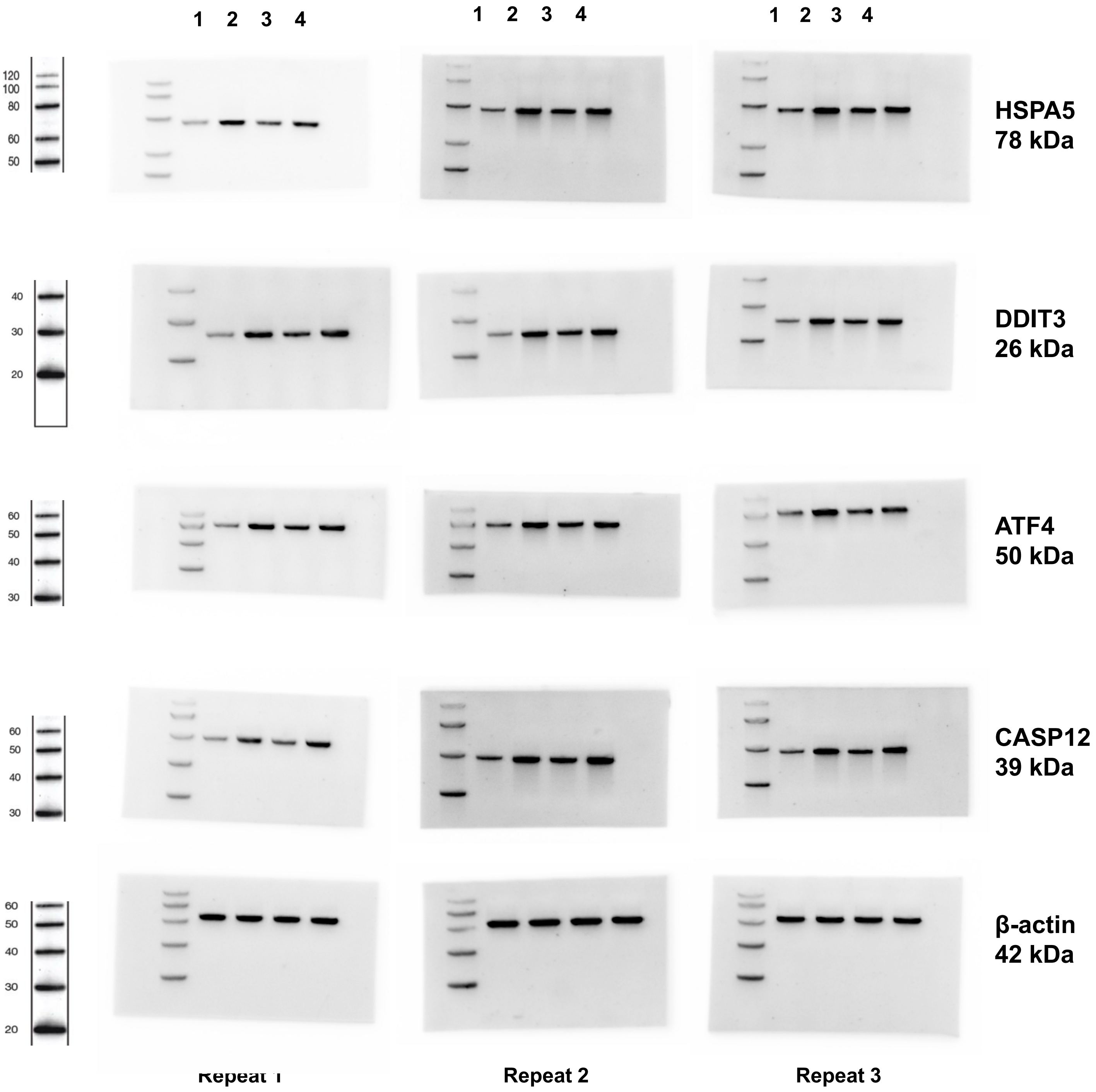

Fig7C

1 Normal

2 AD

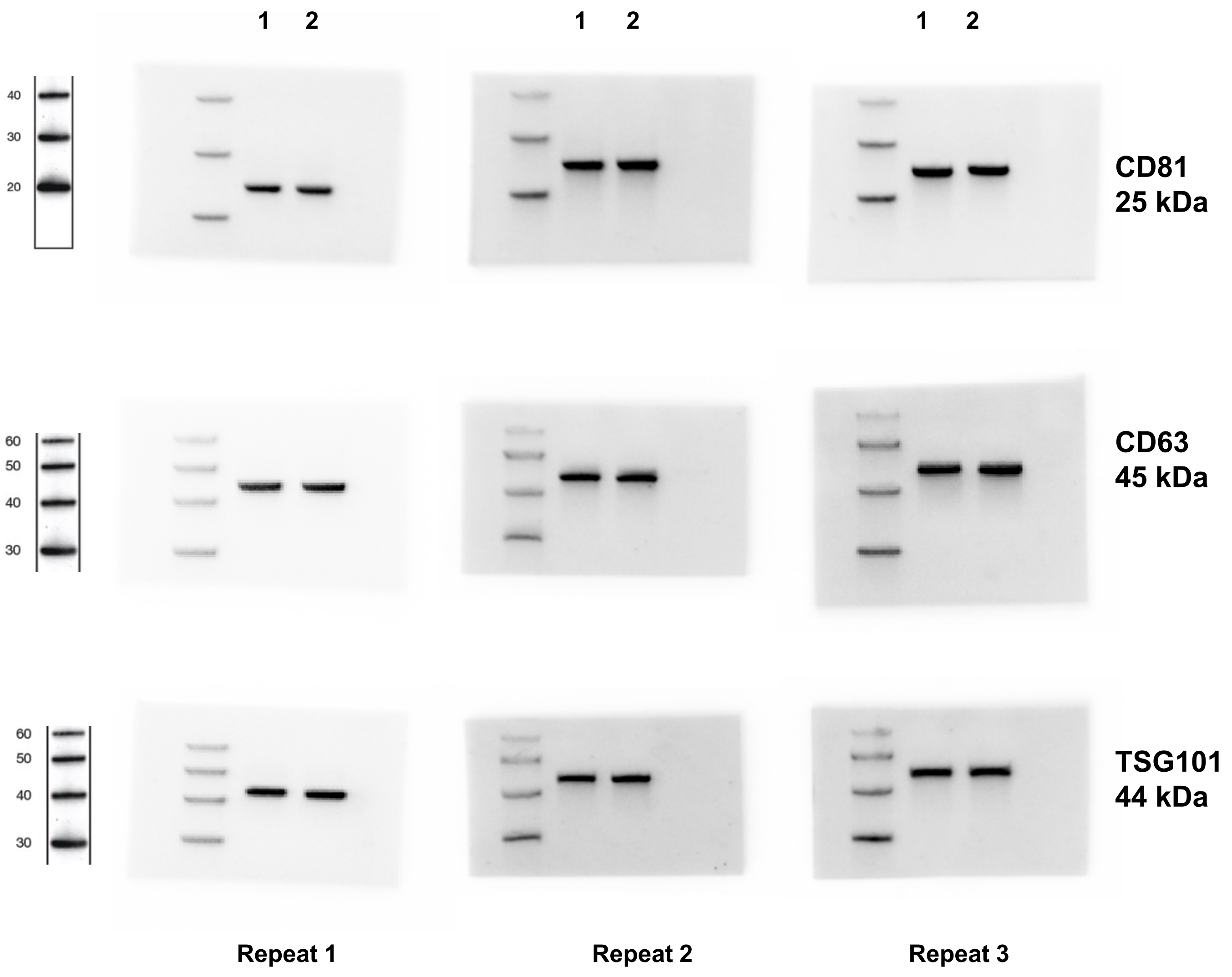

Supplement: Supplementary file 1 — Supplementary file1 (PDF 2149 kb) [file 11064_2022_3563_MOESM1_ESM.pdf]
