# Supplementary figures and images for: CircAXL Knockdown Alleviates Aβ1-42-Induced Neurotoxicity in Alzheimer’s Disease via Repressing PDE4A by Releasing miR-1306-5p
Source: Neurochem Res. 2022 Mar 1;47(6):1707–20. doi: 10.1007/s11064-022-03563-7 (PMC9124172; doi:10.1007/s11064-022-03563-7)

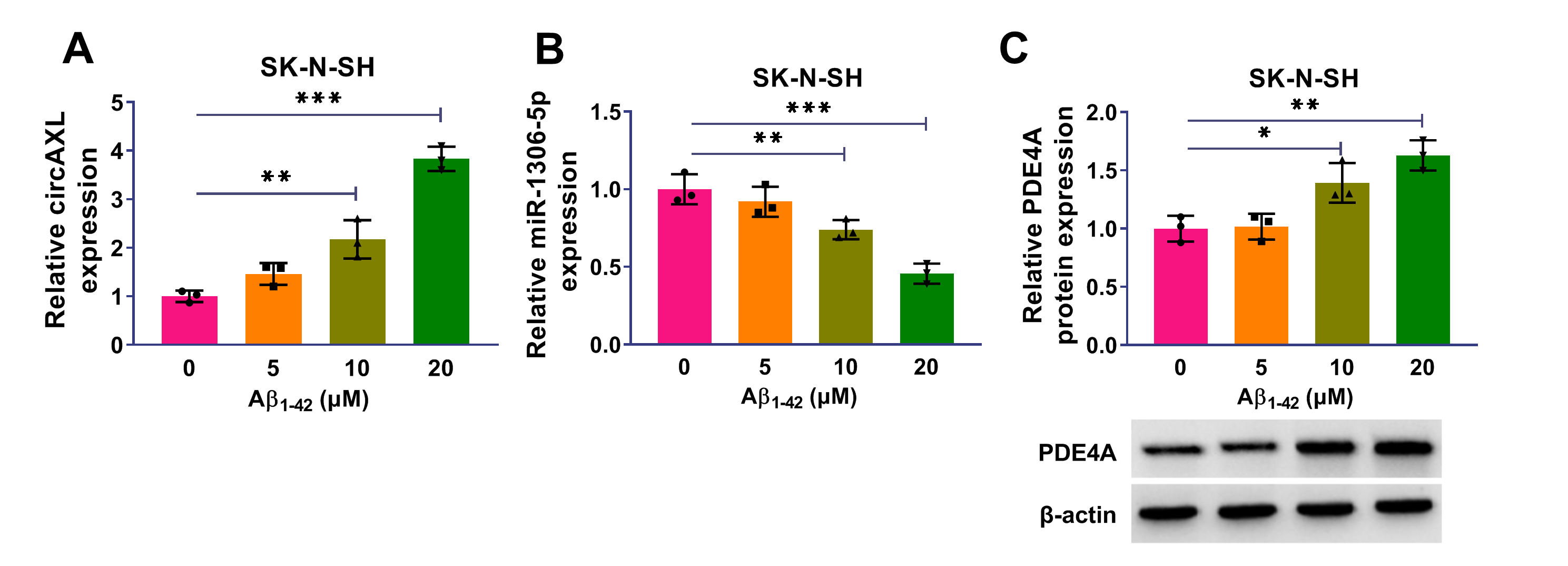

Supplement: Supplementary file 2 — Fig. S1 The expression of circAXL, miR-1306-5p and PDE4A in Aβ1-42-treated SK-N-SH cells. (A) The expression of circAXL was increased in Aβ1-42-treated SK-N-SH cells in a dose-independent manner. (B) The expression of miR-1306-5p was reduced in Aβ1-42-treated SK-N-SH cells in a dose-independent manner. (C) The expression of PDE4A was enhanced in Aβ1-42-treated SK-N-SH cells in a dose-independent manner. *P<0.05, **P<0.01, ***P<0.001. ANOVA (with Tukey’s post-hoc test) was used to analyze the difference. Three independent experiments for these assays were conducted (TIF 374 kb) [file 11064_2022_3563_MOESM2_ESM.tif]
